# *Dlx5* Is a Cell Autonomous Regulator of Chondrocyte Hypertrophy in Mice and Functionally Substitutes for *Dlx6* during Endochondral Ossification

**DOI:** 10.1371/journal.pone.0008097

**Published:** 2009-11-30

**Authors:** Hui Zhu, Andrew J. Bendall

**Affiliations:** Department of Molecular and Cellular Biology, University of Guelph, Guelph, Ontario, Canada; University of Western Ontario, Canada

## Abstract

The axial and appendicular skeleton of vertebrates develops by endochondral ossification, in which skeletogenic tissue is initially cartilaginous and the differentiation of chondrocytes via the hypertrophic pathway precedes the differentiation of osteoblasts and the deposition of a definitive bone matrix. Results from both loss-of-function and misexpression studies have implicated the related homeobox genes *Dlx5* and *Dlx6* as partially redundant positive regulators of chondrocyte hypertrophy. However, experimental perturbations of *Dlx* expression have either not been cell type specific or have been done in the context of endogenous *Dlx5* expression. Thus, it has not been possible to conclude whether the effects on chondrocyte differentiation are cell autonomous or whether they are mediated by *Dlx* expression in adjacent tissues, notably the perichondrium. To address this question we first engineered transgenic mice in which *Dlx5* expression was specifically restricted to immature and differentiating chondrocytes and not the perichondrium. *Col2a1-Dlx5* transgenic embryos and neonates displayed accelerated chondrocyte hypertrophy and mineralization throughout the endochondral skeleton. Furthermore, this transgene specifically rescued defects of chondrocyte differentiation characteristic of the *Dlx5/6* null phenotype. Based on these results, we conclude that the role of *Dlx5* in the hypertrophic pathway is cell autonomous. We further conclude that *Dlx5* and *Dlx6* are functionally equivalent in the endochondral skeleton, in that the requirement for *Dlx5* and *Dlx6* function during chondrocyte hypertrophy can be satisfied with *Dlx5* alone.

## Introduction

The adult vertebrate skeleton appears to be a rather uniform bony tissue. This apparent uniformity belies its embryonic origins, as a mosaic structure originating from diverse progenitors that arise in different germ layers, and its development via two distinct ontological processes: intramembranous and endochondral ossification [1], [2], [3]. In contrast to the direct differentiation of osteoblasts from mesenchymal progenitors that produces the intramembranous bones of the skull and clavicle, the caudal bones of the head, the vertebral column, ribs, and appendicular skeleton develop first as cartilaginous anlagen [4]. Chondroblasts in these skeletal precursors have one of two fates: to undergo a histologically well-defined program of hypertrophy and terminal differentiation in the growing bone or to persist as specialized chondrocytes at the articular surface [5], [6]. The majority of chondroblasts in prospective skeletal elements proliferate in regular columns as radially flattened immature chondrocytes before maturing through three distinct and spatially organized phases: prehypertrophic, hypertrophic, and mineralizing chondrocytes. The hypertrophic chondrocyte differentiation pathway is tightly regulated, with numerous signalling pathways providing both positive and negative signals at each step in the differentiation process. Some of these signals, like Ihh and Delta, are made by subpopulations of differentiating chondrocytes [7], [8], [9]. Others, like members of the TGF-β/BMP and Wnt families, are also made by cells in the perichondrium [10], [11], [12], a tissue that surrounds the growing cartilaginous core and gives rise to the bone collar. Those competing extracellular signals induce or repress transcription factors to regulate chondrocyte proliferation and differentiation; the result is coordinated longitudinal bone growth and joint articulation [6], [13], [14], [15], [16], [17], [18], [19], [20], [21].

*Dlx* homeobox genes encode nuclear transcription factors [22], [23], [24]. In particular, *Dlx5* and *Dlx6* are expressed in all anlagen of the endochondral skeleton. Their expression has been noted in precartilaginous limb bud mesenchyme [25], [26] where each overlaps with expression of *Sox9* [27], [28]. At later stages of skeletogenesis, when proliferating and differentiating chondrocytes are found in spatially distinct regions of the growth plate, *Dlx5* and *Dlx6* are expressed in the post-mitotic prehypertrophic and hypertrophic zones but not in immature chondroblasts in the resting or proliferating zones [27], [28], [29]. *Dlx5* and *Dlx6* are also expressed in the perichondrium/periosteum in the long bones as well as ribs and vertebrae [27], [30], [31], [32]. *Dlx5^−/−^* and doubly deficient *Dlx5/6^−/−^* mice have revealed requirements for *Dlx5* and *Dlx6* during chondrogenesis [28] and chondrocyte hypertrophy [27], [33]. Reciprocally, forced expression of either *Dlx5* or *Dlx6* alone in chicken limb bud micromass cultures stimulated chondrogenesis [28] and misexpression of *Dlx5 in vivo* reduced proliferation of epiphysial chondrocytes and resulted in precocious chondrocyte hypertrophy [27], [29], [34]. Together, these experiments implicate *Dlx5* and *Dlx6* as partially redundant positive regulators of chondrocyte differentiation. However, to date, perturbations of *Dlx* expression have either not been cell type specific or have been done in the context of endogenous *Dlx5* expression. This general concern is particularly germane when seeking to elucidate a cell-autonomous function for *Dlx5* in chondrocyte hypertrophy given endogenous *Dlx5* expression in both the differentiating chondrocytes and in the perichondrium, the site of synthesis of secreted factors that regulate this process in the chondrogenic core. Here, we first describe a transgenic line of mice in which exogenous *Dlx5* expression is targeted to immature chondrocytes using regulatory elements from the *Col2a1* gene. Tissue-specific misexpression of *Dlx5* accelerated chondrocyte hypertrophy and promoted precocious ossification in the endochondral skeleton of these transgenic mice. Visualization of transgene expression in the absence of endogenous *Dlx5* expression (achieved by crossing the allele onto a *Dlx5/6* null background) indicated that transgene expression was limited to the chondrogenic core and not the perichondrium. The subsequent rescue of endochondral ossification defects in *Dlx5/6^−/−^; Col2a1-Dlx5* mice therefore establishes a cell-autonomous function for *Dlx5* during chondrocyte hypertrophy and, furthermore, demonstrates functional equivalence of *Dlx5* and *Dlx6* in the endochondral skeleton.

## Results

### Generation of Transgenic Mice and Characterization of Transgene Expression

**Figure 1:**
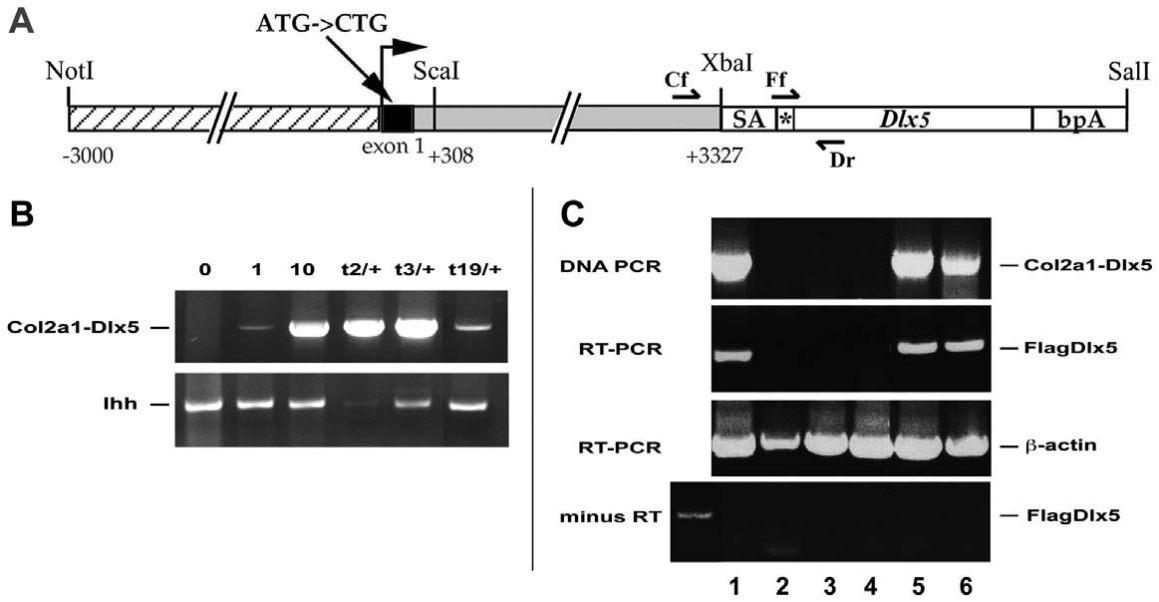
Generation of *Col2a1-Dlx5* transgenic mice. (A) Transgene design. Numbering of promoter and intron elements is with respect to the *Col2a1* transcription start site. The start codon of *Col2a1* in exon 1 has been mutated to prevent translation in this exon [56]. The asterisk indicates the *Flag* sequence 5′ to the murine *Dlx5* open reading frame, bpA, poly-adenylation sequence from the bovine *Growth Hormone* gene; SA, splice acceptor. Half arrows indicate the approximate location of primers for genotyping: Col2a1 forward (Cf) plus Dlx5 reverse (Dr), and for RT-PCR: Flag forward (Ff) plus Dr. (B) Transgene copy number. Semi-quantitative PCR was used to compare the approximate transgene copy number in transgenic founder mice: t2/+ and t3/+ represent dead hemizygous founders and t19/+ is a hemizygote neonate from the stable *Col2a1-Dlx5^t19/+^* line. Control lanes from the left are: wild type DNA (0), wild type DNA mixed with *p3000i3020Col2a1-Dlx5* at 1 copy per genome equivalent (1) or 10 copies per genome equivalent (10). Amplification of a genomic fragment of the single copy gene *Ihh* was used to judge relative amplification of the transgene. (C) Specific amplification of expressed *Flag-Dlx5* sequence from transgenic embryos in a reverse transcriptase-dependent manner. RT-PCR analysis of five F4 generation embryonic day (E) 17.5 embryos from a *wt* x *Col2a1-Dlx5^t19/+^* mating demonstrates stable heritable expression of the *Col2a1-Dlx5* transcription unit. The first lane in the bottom panel (+) shows a positive PCR control for the minus RT experiment. Non-adjacent lanes from the same gel have been spliced together to generate the figure. Lane numbers refer to individual embryos.

*Dlx5* and its *cis*-linked paralogue *Dlx6* function as positive regulators of both chondrogenesis and chondrocyte hypertrophy in the endochondral skeleton [27], [28], [29], [33]. To further investigate the function of *Dlx5* during chondrocyte hypertrophy *in vivo*, we generated transgenic mice in which a *Dlx5* cDNA was expressed under control of the promoter and intron 1 enhancer of the *Col2a1* gene (Fig. 1A) so as to target *Dlx5* expression to immature chondrocytes following their differentiation from pluripotent mesenchymal precursors. Following pro-nuclear injection, we obtained four *Col2a1-Dlx5* transgenic founders; these four founders (or their hemizygous offspring) had a variable number of copies of the transgene, as measured by semi-quantitative PCR from genomic DNA (Fig. 1B). The three founders with the highest number of copies (more than ten) were recovered dead as neonates and their transgenic alleles are referred to as *Col2a1-Dlx5^t1^*, *Col2a1-Dlx5^t2^*, and *Col2a1-Dlx5^t3^*. The viable founder with allele *Col2a1-Dlx5^t19^* had fewer than four copies as a hemizygote and was used to establish a stable transgenic line expressing epitope-tagged *Dlx5* (Fig. 1C).

**Figure 2:**
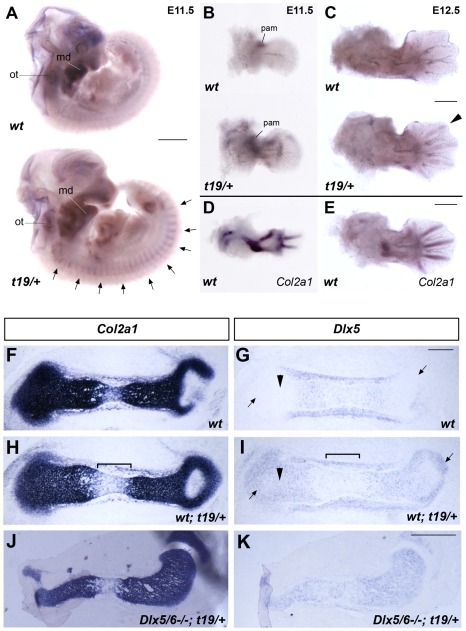
Transgene expression in the endochondral skeleton. (A–E) Whole mount *in situ* hybridization of *Dlx5* (A–C) or *Col2a1* (D,E) probes to wild type (*wt*) and *Col2a1-Dlx5^t19/+^* transgenic (*t/+*) littermates at E11.5 and E12.5. Arrows point to somites in panel A. Arrowhead points to digit 2 in panel C. (F–I) Section *in situ* hybridization of *Col2a1* (F,H) or *Dlx5* (G,I) riboprobes to adjacent sections of the femur of wild type (F,G) or *Col2a1-Dlx5^t19/+^*transgenic embryos (H,I) at E14.5. Arrowheads indicate the *Col2a1*-positive proliferating zones, arrows point to the resting zones, and the bracket demarcates the hypertrophic zone in panels G and I. Proximal is to the right. (J,K) Section *in situ* hybridization of *Col2a1* (J) or *Dlx5* (K) riboprobes to adjacent sections of the tibia of *Dlx5/6^−/−^*; *Col2a1-Dlx5^t19/+^* transgenic embryos at E14.5. Proximal is to the right. md, mandibular arch; ot, otic vesicle; pam, proximal anterior mesoderm. Scale bar = 1 mm in A, 0.5 mm in B–E,J,K, 0.2 mm in F–I.

To examine the tissue distribution of transgene expression, we examined embryos following whole mount *in situ* hybridization with a *Dlx5* riboprobe. *Dlx5* expression was visualized in a number of locations that normally express the definitive chondroblast marker *Col2a1* (Fig. 2). In particular, and in contrast to non-transgenic littermates, *Dlx5* expression was apparent in somites along the entire rostro-caudal axis and in rib cartilage and throughout the skeletal anlagen of the limb, where it closely matched expression of the endogenous *Col2a1* gene in the stylopod and zeugopod (Fig. 2A–E). In contrast, at E12.5 ectopic expression of *Dlx5* was most obvious in the phalanges, where it coincided with endogenous *Col2a1* expression. Endogenous expression of *Dlx5* in the otic vesicle, mandibular arch, branchial arches 2 and 3, or in a proximal anterior mesodermal domain in the limb was not altered in transgenic embryos. To confirm that *Dlx5* was being expressed ectopically in immature chondroblasts, we examined tissue sections following *in situ* hybridization. Indeed, *Dlx5* was expressed throughout the *Col2a1*-expressing zones of the long bones and vertebrae (Fig. 2F–I and data not shown), including the resting and proliferating zones of the long bone epiphyses, where its expression is not usually detectable (compare Fig 2H,I). Moreover, in the hypertrophic zone, where *Col2a1* transcription is normally down regulated, we saw a parallel decrease in *Flag-Dlx5* transcript abundance (compare Fig. 2G,I). To view transcription of the transgene in the absence of endogenous *Dlx5* expression, we introduced the *Col2a1-Dlx5^t19^* allele into a *Dlx5/6^−/−^* background. Section *in situ* hybridization to the long bones of the limbs of *Dlx5/6^−/−^*; *Col2a1-Dlx5^t19/+^* embryos revealed that transgene expression was restricted to the cartilaginous core of the skeletal anlagen and was not expressed in the surrounding perichondrium (Fig. 2J,K). In summary, expression of the *Col2a1-Dlx5^t19^* allele faithfully replicated endogenous *Col2a1* gene expression in chondrocytes and resulted in ectopic *Dlx5* expression in immature and proliferating chondroblasts.

### Acceleration of Chondrocyte Hypertrophy Following Forced Expression of *Dlx5* in Chondroblasts

**Figure 3:**
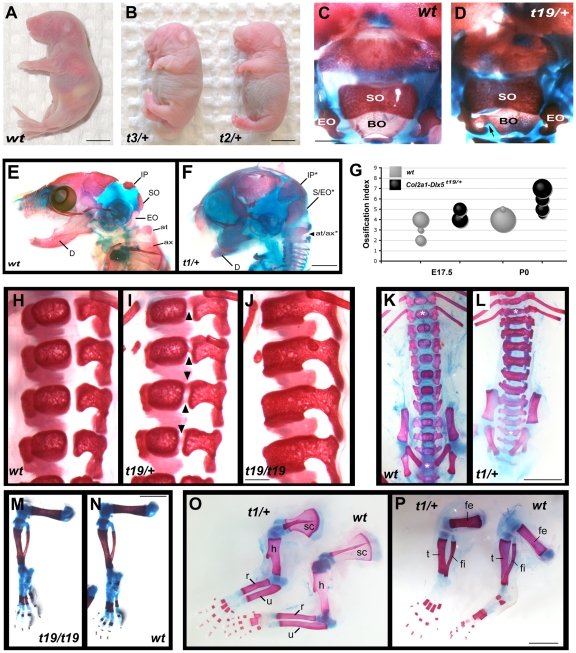
Dose-dependent hypermineralization in the endochondral skeleton of *Col2a1-Dlx5* transgenic neonates. (A,B) Lateral views of a wild type CD-1 P0 pup (A) and two hemizygous *Col2a1-Dlx5* transgenic pups (B), both found dead shortly after birth. (C,D) Dorsal view of the caudal skull of wild type (C) and *t19/+* (D) neonates at postnatal day zero (P0) following staining with alcian blue and alizarin red. The arrow points to an area of fusion between the basioccipital and exoccipital bones in panel D. (E,F) Lateral views of a wild type (E) and *t1/+* transgenic founder (F) at P0 after staining with alcian blue and alizarin red. (G) Bubble graph of the ossification index of multiple litters of *t19/+* hemizygotes and their wild type littermates. Following staining with alcian blue and alizarin red, a score was assigned to each embryo or neonate that reflected the extent of occipital ossification relative to a typical wild type at that stage: 1 = no brain case (exencephalic), 2 = brain case intact but no ossification of the supraoccipital (SO) apparent, 3 = smaller SO than is seen in a majority of wild type specimens (which were assigned a score of 4); 5 = obviously smaller distance between the SO and exoccipital (EO), or between the EO and the basioccipital (BO), compared to a majority of wild type; 6 = unilateral touching of SO and EO or of EO and BO; 7 = bilateral contact between SO and EO, or between the EO and the BO, or fusion of any of these bones. Bubble size is proportional to the number of neonates with a given score; the smallest circles represent a single individual, the largest circle represents 17 individuals. (H–J) Ventral views of wild type (H), hemizygous (I) and homozygous (J) embryos with the *Col2a1-Dlx5^t19^* allele at P1 following alizarin red staining. Arrowheads point to precociously mineralized vertebrae in panel I. (K,L) Ventral views of wild type (K), and *Col2a1-Dlx5^t1/+^* transgenic founder (L) at P0 following alcian blue and alizarin red staining. The most caudal thoracic (T13) and sacral (S4) vertebrae are marked with white asterisks. (M–P) Limb skeleton preparations from neonates with the genotypes as shown following alcian blue and alizarin red staining. at, atlas; at/ax*, fused atlas and axis; ax, axis; BO, basioccipital; D, dentary, EO, exoccipital; fe, femur; fi, fibula; h, humerus; IP, interparietal; IP*, interparietal bone with expanded mineralization; r, radius; sc, scapula; SO, supraoccipital; S/EO*, fused supraoccipital and exoccipital bones; t, tibia; u, ulna; wt, wild type. Scale bar = 5 mm in A,B, 1 mm in C,D, 2 mm in E,F,K–P, 0.5 mm in H–J.

To examine the functional consequences of misexpressing *Dlx5* in chondrocytes, we first examined skeletal preparations of neonates at postnatal day zero (P0) or P1 after staining cartilage and mineralizing bone with alcian blue and alizarin red respectively. All three dead founders, or viable neonates bearing the *Col2a1-Dlx5^t19^* allele, examined this way had a common phenotype of hypermineralization of the endochondral skeleton (Fig. 3). In its most severe form, seen in three non-viable founders, hypermineralization resulted in shorter stature (compare Fig. 3A, B). The extent of hypermineralization, as estimated from the extent of alizarin red staining and generalized skeletal dysmorphology, followed the allelic series *Col2a1-Dlx5^t19/+^* < *Col2a1-Dlx5^t19/t19^* < *Col2a1-Dlx5^t2/+^*, *Col2a1-Dlx5^t3/+^* < *Col2a1-Dlx5^t1/+^*, and correlated with our estimates of transgene copy number (Fig. 1B). We analyzed each component of the skeleton in turn and uncovered a differential sensitivity to transgene expression in the axial and appendicular skeleton.

The basioccipital, exoccipital and supraoccipital bones of the rostral skull arise from the most cranial somites [35]. Eventually, the occipital bones fuse to surround the foramen magnum but the mineralizing occipital bones of neonates are normally well separated at birth, with alcian blue-staining cartilaginous matrix between the supraoccipital and exoccipital bones and between the exoccipital and basioccipital bones (Fig. 3C). *Col2a1-Dlx5^t19/+^* hemizygotes showed variable degrees of expanded mineralization of the supra- and basioccipital bones at birth, such that these bones sometimes touched or were fused at discrete points with the adjacent edge of an exoccipital bone (Fig. 3D). The degree of occipital ossification was scored for wild type (n = 29) and *t19/+* hemizygotes (n = 35) at two developmental stages and is shown, along with an explanation of the scoring system, in Fig. 3G. By P0, all transgenic pups examined (n = 24) showed some degree of advanced ossification in the occipital bones, compared to 1 of 18 wild type neonates. In its most severe form, as exemplified by the *Col2a1-Dlx5^t1/+^* founder, this hypermineralization was so excessive that the supraoccipital and exoccipital bones were completely fused, as were the first two cervical vertebrae and there was hypermineralization in the chondrocranium surrounding the interparietal bone (Fig. 3F). Indeed, the entire head was dysmorphic in the recovered *t1/+*, *t2/+*, and *t3/+* founders: deeper dorso-ventrally but foreshortened rostro-caudally with pronounced shortening of the maxillary process and dentary such that the tongue protruded well past the jaws. Non-endochondral components of the skull like the dentary and frontonasal bones were likely to have been secondarily affected by accelerated mineralization in the chondrocranium and were similarly affected in all three dead founders (Fig. 3A,B and E,F).

The axial skeleton originates in the somitic sclerotome and vertebrae with a diversity of shapes and functions subsequently form along the rostral-caudal axis. Adjacent somites contribute to single vertebral anlagen, which assemble from three sclerotomal compartments: dorso-medial (dorsal neural arch and spinous process), lateral (laminae and pedicles of the neural arch and ribs at thoracic levels), and ventral (centrum and vertebral discs). Embryonic vertebrae have discrete ossification centres in the centrum and the neural arches, which normally fuse post-natally. The vertebrae of P1 *Col2a1-Dlx5^t19/+^* hemizygous neonates (n = 8) showed precocious ossification between these centers in the form of narrow bridges of mineralized tissue that joined the ossified centrum and neural arch. This precocious ossification was further advanced in *Col2a1-Dlx5^t19/t19^* homozygotes (n = 9) such that the majority of centra were completely fused to the adjacent neural arches (Fig. 3H–J). The overall morphology of vertebrae and length of the vertebral column were not strongly affected in this line. While all vertebrae of the *Col2a1-Dlx5^t1/+^* founder were completely fused, vertebrae were additionally dysmorphic and the vertebral axis was significantly shorter in the strongest phenotypes (Fig. 3K,L), resulting in the short stature of these founders (Fig. 3A,B).

In contrast to the axial skeleton, the limbs of *Col2a1-Dlx5* neonates were mildly affected. *Col2a1-Dlx5^t19/+^* limbs were indistinguishable from wild type in both overall size and in the extent of mineralization. The limbs of homozygous *Col2a1-Dlx5^t19/t19^* neonates averaged slightly smaller than wild type littermates (e.g. femurs from homozygotes averaged 94% of wild type femur length, n = 4, Fig. 3M,N) but this was not statistically significant. A limb phenotype became more obvious in the most severely affected founder (*Col2a1-Dlx5^t1/+^*) in which the long bones were shorter and thicker than non-transgenic controls, particularly in the hindlimb, and the relative extent of mineralization was more advanced in the femur compared to wild type controls (Fig. 3O,P). Finally, we did not observe truly ectopic mineralization in cartilaginous structures that do not normally ossify, like the trachea or chondrocostal cartilage. In conclusion, our observations of the skeletons from four independent *Col2a1-Dlx5* alleles demonstrate that misexpression of *Dlx5* in immature chondrocytes promoted endochondral ossification in a dose dependent manner and that, above a certain threshold, this resulted in neonatal lethality.

**Figure 4:**
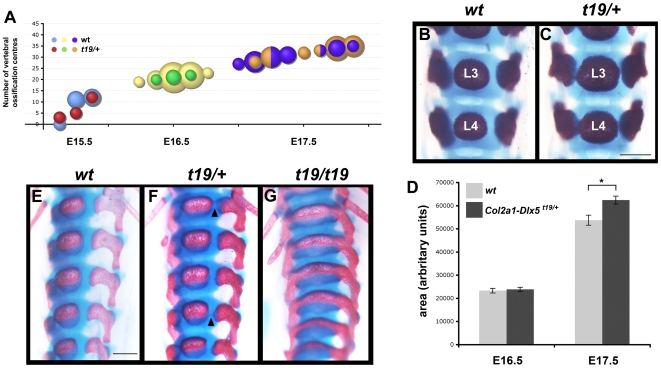
The timing of the onset of mineralization in the vertebral bodies was not affected by *Dlx5* but the subsequent rate of ossification was. (A) Bubble graph depicting the number of vertebral centra in which mineralization was detectable after alcian blue and alizarin red staining of wild type and t19/+ hemizygous embryos at three developmental stages. The smallest bubble represents a sample of one, the largest represents eight individuals. (B,C) Dorsal view of the third and fourth lumbar vertebrae of a wild type and *t19/+* hemizygote at E17.5 following alcian blue and alizarin red staining. (D) Quantitation of the area of the L3 centrum that had mineralized, plotted as average area ± sem. Wild type, white bars (n = 17 and n = 18 at E16.5 and E17.5 respectively); *Col2a1-Dlx5^t19/+^*, grey bars (n = 4 and n = 13 at E16.5 and E17.5 respectively); * *P*<0.005. (E–G) Ventral views of wild type (E), hemizygous (F) and homozygous (G) embryos with the *Col2a1-Dlx5^t19^* allele at E17.5 following alizarin red staining. Arrowheads point to precociously mineralizing vertebrae in panel F. Scale bar = 0.5 mm for all photomicrographs.

We next asked whether the timing of onset of mineralization was affected in *Col2a1-Dlx5* embryos. At E14.5 there was no mineralization in the centra of either wild type or transgenic littermates, as visualized by alizarin red staining (not shown). Thereafter, the initiation of mineralization in the centrum at a specific axial level in wild type embryos was somewhat variable such that, by E15.5, the number of vertebral centra in which mineralization had commenced was quite disparate among littermates of the same genotype, varying from zero to twelve (Fig. 4A). While this variability continued through the next two days of development, we reasoned that, if *Dlx5* were affecting the onset of mineralization, transgenic embryos would have consistently more ossification centres in their vertebral centra at a given developmental stage. This was not the case however (Fig. 4A). We also measured the proportion of the centrum that had mineralised at different times to ask whether, once initiated, endochondral ossification was accelerated. By E16.5, mineralization was well under way in all thoracic and lumbar vertebrae in all embryos, regardless of genotype. We found no difference in the extent of mineralization at a given axial level at this stage when comparing transgenic or wild type littermates (Fig. 4D). By E17.5 however, there was a clear difference in the extent of ossification of the vertebral bodies in *Col2a1-Dlx5^t19/+^* hemizygotes and additional areas of mineralization were first apparent that bridged the mineralising region of the centrum with mineralised tissue in the neural arches (Fig. 4B–F). In contrast to hemizygotes at E17.5, when bridges were faintly detectable in a variable number of vertebrae, all vertebrae from the atlas to the fourth sacral vertebrae were affected in *t19/t19* homozygotes and, moreover, the two mineralization centres were completely fused (Fig. 4G). We also measured ossification of the ribs and found a 10% difference in the extent of ossification of the ribs at P0 (*P*<0.05, n = 9 wild type, n = 12 transgenic). However, no significant difference in the extent of ossification in the long bones of the limbs was apparent up to P0 (data not shown, n = 20 wild type, n = 12 transgenic). Thus, expression of *Dlx5* in somitic cartilage did not appear to affect the initial timing of mineralization, but rather contributed to a dose-dependent acceleration of ossification once initiated.

**Figure 5:**
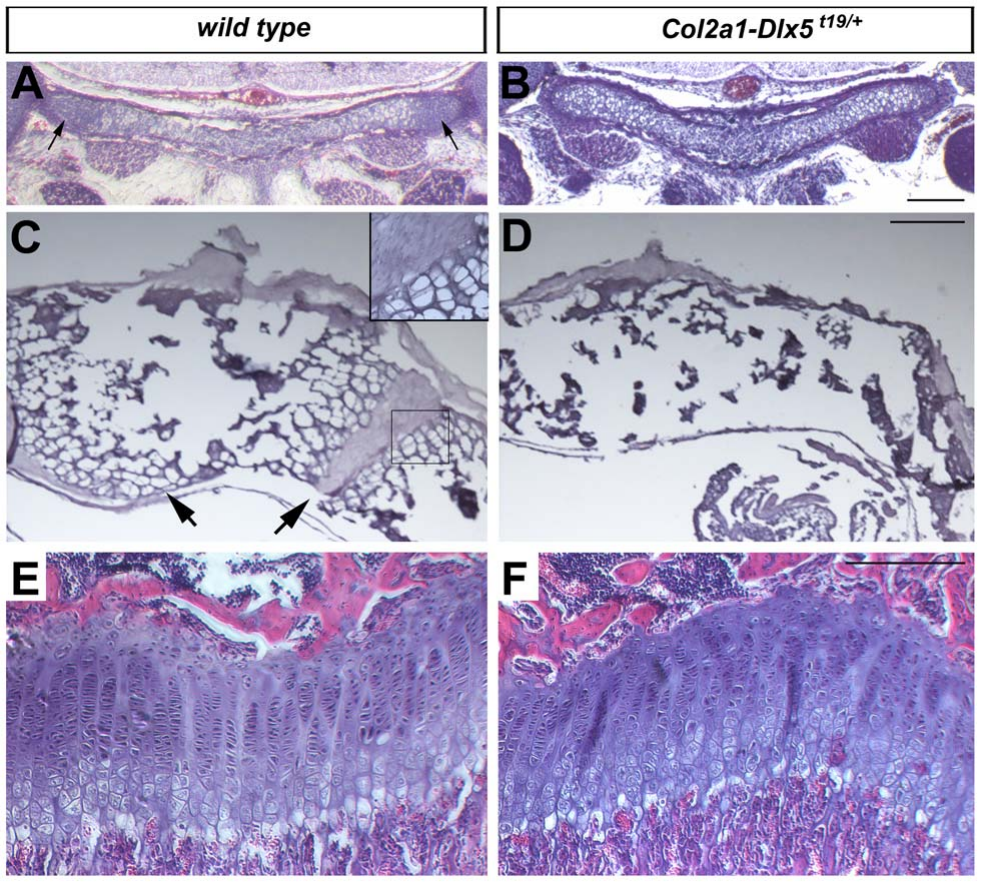
Misexpression of *Dlx5* in chondrocytes accelerated chondrocyte hypertrophy. (A,B) Haematoxylin and eosin (H&E) staining of coronal sections of the head of wild type (A) and *Col2a1-Dlx5* transgenic (B) E16.5 embryos. Arrows point to proliferating zones in panel A. Dorsal is up. (C,D) Alizarin red-stained and cleared vertebral columns of wild type (C) and transgenic (D) P1 embryos were embedded in paraffin and sectioned. Arrows point to cartilaginous tissue in panel C. Boxed region is shown at higher magnification in panel C insert. Dorsal is up. (E,F) H&E stained longitudinal sections through the proximal humerus of wild type (E) and transgenic littermate (F). Proximal is up. Scale bar = 0.2 mm in A,B,E,F, 2 mm in C,D.

We next sought to determine whether precocious mineralization of the endochondral skeleton was due to an underlying acceleration of chondrocyte differentiation. We examined hematoxylin and eosin stained sections through the head, trunk and limbs at various stages (Fig. 5). At E16.5, before a mineralization phenotype was apparent in the caudal skull, advanced hypertrophy of the basioccipital bone was apparent. The lateral edges of the basioccipital bone contain small, rounded chondroblasts in wild type embryos, whereas hypertrophic chondrocytes occupied the entire element in transgenic littermates (Fig. 5A,B). Transverse sections through the vertebrae of *Col2a1-Dlx5^t19/+^* transgenic neonates confirmed that mineralization had occurred throughout the vertebrae, whereas the ossification centres of the centrum and neural arches of wild type pups were separated by blocks of cartilaginous tissue that contained both radially flattened and hypertrophic chondrocytes (Fig. 5C,D). Finally, we examined the growth plates of three-week-old mice after H&E staining. There were no gross distortions in the overall architecture of the growth plates of transgenic weanlings (n = 3) compared to wild type littermates (n = 4), with both proliferating and hypertrophic zones being of similar size, although dividing chondrocytes in the proliferative zone were stacked somewhat less regularly in transgenic mice (Fig. 5E,F). Thus, forced expression of *Dlx5* in immature chondrocytes resulted in accelerated chondrocyte hypertrophy in the axial skeleton and a more subtle effect in the growth plate of limbs.

**Figure 6:**
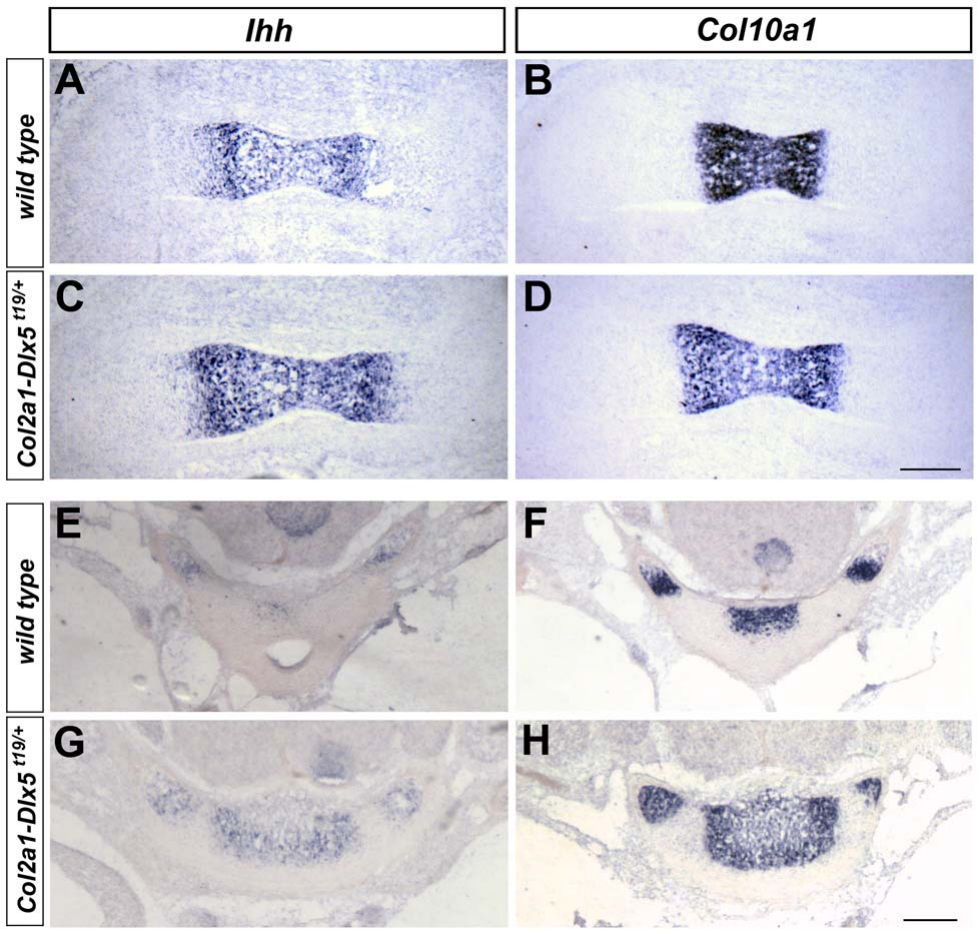
Expansion of prehypertrophic and hypertrophic marker gene expression in *Col2a1-Dlx5* transgenic mice. (A–D) *In situ* hybridization of *Ihh* (A,C) or *Col10a1* (B,D) riboprobes to adjacent cryosections of wild type and *Col2a1-Dlx5* transgenic femurs at E14.5. Proximal is to the right. (E–H) *In situ* hybridization of *Ihh* (E,G) or *Col10a1* (F,H) riboprobes to adjacent cryosections of wild type and *Col2a1-Dlx5* transgenic vertebral centra at E16.5. Dorsal is up. Scale bar = 0.2 mm for all photomicrographs.

To further confirm the basis of the phenotype, we examined the expression of markers of prehypertrophic and hypertrophic chondrocytes in transverse sections through the limbs and vertebrae at times before an overt effect on mineralization was apparent. Consistent with the idea that *Dlx5* promoted precocious ossification via accelerated chondrocyte hypertrophy, expression of both prehypertrophic (*Ihh*) and hypertrophic (*Col10a1*) markers was expanded in the limbs and vertebrae of *Col2a1-Dlx5^t19/+^* embryos compared with wild type littermates (Fig. 6). In summary, expression of *Dlx5* in immature chondrocytes promoted chondrocyte hypertrophy and endochondral ossification.

### The Chondrocyte Hypertrophy Function of *Dlx5* Is Cell Autonomous and Is Sufficient to Rescue Endochondral Ossification in *Dlx5*/6 Null Embryos

**Figure 7:**
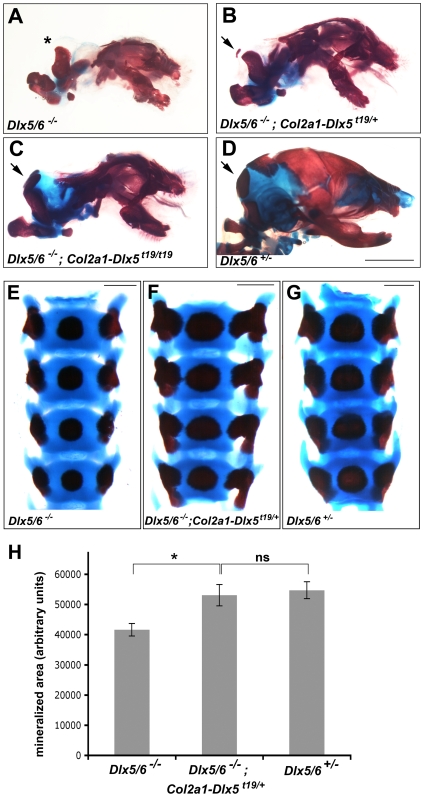
Chondrocyte-specific expression of *Dlx5* rescued endochondral defects in *Dlx5/6* null embryos. (A–D) Lateral views of the heads of P0 neonates with the genotypes shown after staining with alcian blue and alizarin red. Asterisk indicates a missing supraoccipital bone in panel A. Arrows point to supraoccipital bones in panels B–D. (E–G) Dorsal view of the second to fifth lumbar vertebrae from a *Dlx5/6^−/−^* (E), *Dlx5/6^−/−^*; *Col2a1-Dlx5^t19/+^* (F), and *Dlx5/6^+/−^* (G) embryo at E17.5 following alcian blue and alizarin red staining. Since *Dlx5/6^−/−^* embryos are smaller, digital images of L2 to L5 at E17.5 were scaled to the same vertebral size to allow measurements of the relative mineralization of the vertebral bodies. Rostral is at the top. (H) Quantitation of mineralization in the L3 centrum of *Dlx5/6^−/−^* (n = 9), *Dlx5/6^−/−^; Col2a1-Dlx5^t19/+^* (n = 3), and *Dlx5/6^+/−^* (n = 9) vertebrae, plotted as average area ± sem. * *P*<0.05; ns, *P*>0.05. Scale bar = 2 mm in A–D, 0.5 mm in E,F,G.

*Dlx5* and *Dlx6* have shared essential functions in mandibular patterning and AER perdurance and *Dlx5/6* null neonates are exencephalic as a result of a failure to ossify the skull vault; in particular, *Dlx5/6* neonates lack supraoccipital and interparietal bones at birth [33], [36], [37], [38]. Targeted deletion of both *Dlx5*/*6* also revealed shared functions in the endochondral skeleton. Defects that arise as a result of delayed or absent chondrocyte differentiation vary from the complete absence of mineralised endochondral elements, notably the paired supraoccipital bones, to delayed mineralization throughout the vertebral and appendicular skeleton [33], [36]. While the limb and axial skeleton of *Dlx6^−/−^* mice has not yet been described, *Dlx6* is largely redundant with *Dlx5* in patterning the first branchial arch [39]. Further understanding of the functional equivalence of *Dlx5* and *Dlx6* in endochondral ossification requires the substitution of one for the other in an *in vivo* context. We asked whether cartilage-specific expression of *Dlx5* could rescue cartilage maturation defects in *Dlx5/6* null embryos by crossing *Col2a1-Dlx5^t19^* hemizygous mice (CD-1 background) with heterozygous *Dlx5/6^+/−^* mice (C57Bl/6 x DBA background) to specifically reconstitute *Dlx5* function in the cartilage of otherwise *Dlx5/6* null embryos. *Dlx5/6^−/−^*; *Col2a1-Dlx5^t19/+^* neonates showed a rudiment of a mineralized element of varying size, located dorsal to the exoccipital bones, which was interpreted to be supraoccipital in identity (Fig. 7B, n = 4), and partial rescue of the supraoccipital was sometimes unilateral. In contrast, *Dlx5/6^−/−^*; *Col2a1-Dlx5^t19/t19^* homozygotes had mineralised supraoccipital bones that were of a similar size to those in *Dlx5/6^+/−^* or *wild type* littermates (Fig. 7C,D, n = 3). Notably, the interparietal, which forms via intramembranous ossification, was not rescued in these animals, nor was transformation of mandibular structures to a maxillary identity (Fig. 7). Finally, we asked whether rescue of endochondral ossification was a general feature of these embryos by examining a more quantitative trait, namely ossification of the vertebral centra. Ossification of the centra of *Dlx5/6* null embryos lags that in wild type littermates, being, on average, 76% of that in heterozygous or wild type littermates at E17.5 (Fig. 7E–H). Vertebral mineralization in *Dlx5/6^−/−^*; *Col2a1-Dlx5^t19/+^* embryos, however, was indistinguishable from *Dlx5/6^+/−^* littermates. Taken together, our data show that *Dlx5* can fully compensate for *Dlx6* in the endochondral skeleton. While we cannot formally exclude the possibility that *Dlx5* was expressed at very low levels in the perichondrium, that were below our detection limits, our results are consistent with a cell autonomous function for *Dlx5* during chondrocyte hypertrophy.

## Discussion

### *Dlx5* and *Dlxt6* as Cell Autonomous Regulators of Chondrocyte Hypertrophy

We have generated a line of transgenic mice in which the transcription factor *Dlx5* is expressed in proliferating chondroblasts. Interestingly, the level of expression of exogenous *Dlx5* was similar to that of endogenous *Dlx5*, as measured by *in situ* hybridization, with both being expressed at much lower levels than *Col2a1*. Our finding is consistent with the observations of others, using essentially the same *Col2a1* sequences [40] and likely reflects the absence of *cis*-acting sequences that contribute to the higher level expression of *Col2a1*. We consider this to be an advantage of our mouse model since ectopic expression of *Dlx5* is at near physiological levels in *Col2a1-Dlx5^t19^ hemizygotes.* Through cell type-specific manipulation of its expression, we provide evidence that *Dlx5* is required cell autonomously for chondrocyte hypertrophy and that *Dlx6* has a redundant function in this tissue. This is best demonstrated by the fact that restoration of *Dlx5* expression in chondrogenic condensations in the chondrocranium was sufficient to rescue the supraoccipital bones in *Dlx5/6*-deficient mice. Rescue of the supraoccipital bones in *Dlx5/6^−/−^*; *Col2a1-Dlx5^t19/t19^* neonates further indicates that chondrogenic differentiation events prior to activation of *Col2a1* are not defective in *Dlx5/6* null embryos but rather that the absence of the supraoccipital bones in *Dlx5/6^−/−^* neonates is due to a subsequent block in chondrocyte differentiation. This chondrogenic function is apparently independent of the role of *Dlx5* or *Dlx6* as osteoblast differentiation genes or as regulators of craniofacial patterning since *Dlx5/6^−/−^*; *Col2a1-Dlx5^t19/t19^* neonates retained intramembranous bone defects and transformation of mandibular to maxillary structures. Similarly, *Dlx5* likely has an independent function in the perichondrium where it may promote differentiation to the periosteum. Another study, in which *Dlx5* was specifically misexpressed in chondrocytes, documented enhanced chondrocyte hypertrophy in the limb [34]. These tissue-specific manipulations of *Dlx5* expression validate the results of more generalized over-expression studies with this gene [29].

*Dlx6^−/−^* mice display a range of first branchial arch defects that closely resemble those seen in *Dlx5^−/−^* neonates, but that are milder [39]. The non-additive nature of the patterning defects that occur following the combined deletion of *Dlx5* and *Dlx6* suggest functional redundancy of these genes in the first branchial arch. Similarly, gain-of-function studies point to a quantitatively equivalent function in stimulating multipotent precursors to differentiate into chondroblasts [28]. The functional equivalence of these genes in a specific tissue had not previously been addressed *in vivo*; a definitive test of functional equivalency requiring substitution of *Dlx5* coding sequences for those of *Dlx6*. This requirement is satisfied in embryos with combined *Col2a1-Dlx5^t19^* and *Dlx5/6^−/−^* alleles and constitutes a test of functional equivalency in cartilage. That both quantitative and qualitative defects in endochondral ossification were rescued by chondrocyte-specific expression of *Dlx5* argues that *Dlx5* and *Dlx6* are functionally interchangeable in chondrocytes and that specific endochondral elements depend on different levels of Dlx5 or Dlx6 activity; vertebral ossification was not completely dependent on either *Dlx5* or *Dlx6* (since it was not completely blocked in *Dlx5/6* null embryos) and was rescued to wild type levels in hemizygous *Col2a1-Dlx5* embryos while complete rescue of the supraoccipital bones required homozygosity of the transgene in a Dlx5/6 null background. Nonetheless, it is quite likely that some functions of the *Dlx5-Dlx6* locus will depend on diverged protein functions; some genes behave differently following loss of *Dlx6* versus *Dlx5* in the mandibular arch, for example [39]. This is not altogether surprising given the striking differences in the amino acid sequences of the Dlx5 and Dlx6 proteins and the differential distribution of their transcriptional activation activities [28].

### A Hierarchy of Transcription Factors in Chondrocyte Differentiation

Like *Dlx5*, *Runx2* (encoding two isoforms that differ at their amino-termini) is a multifunctional regulator of both chondrocyte and osteoblast differentiation [41] and the two factors have some remarkable parallels in both expression and function. Like *Dlx5*, *Runx2* is expressed in cartilaginous condensations and later, during long bone growth, both genes are expressed in prehypertrophic and hypertrophic chondrocytes. In addition, *Dlx5* and *Runx2* are expressed in the perichondrium flanking the prehypertrophic and hypertrophic zones and, in both cases, perichondrial expression extends over the proliferating zone ([27], [40], [42], [43], [44], [45], [46], [47] and see Fig. 2H). As this study has demonstrated for *Dlx5*, either *Runx2* isoform accelerated chondrocyte hypertrophy in the axial and appendicular skeleton when misexpressed in immature cartilage [40], [48]. Isoform selective deletion further revealed that *Runx2-II* has a non-redundant function in chondrocyte maturation [49] and *Runx2-II*, rather than *Runx2-I*, is specifically expressed in prehypertrophic and hypertrophic chondrocytes [47]. *Runx2* isoforms also induced ectopic chondrocyte hypertrophy in transgenic mice, in which persistent cartilage in the trachea and chondrocostal cartilage was diverted to a hypertrophic fate [40], [48], and retroviral-mediated misexpression of *Runx2-II* converted persistent cartilage to a hypertrophic fate in the chicken hyoid skeleton [50]. In contrast, *Dlx5* appears unable to induce ectopic mineralization. None of our four *Col2a1-Dlx5* alleles caused ectopic mineralization in persistent cartilage. Even in tissues that normally mineralise, *Dlx5* misexpression did not appear to affect the timing of the initial appearance of a calcified matrix, but rather contributed to a subsequent acceleration once begun. Indeed, in the vertebrae, the effects of an accelerated deposition of calcified matrix in the centra were not apparent until two days after mineralization was first visible. Similarly, we never observed isolated patches of mineralized vertebral tissue between the centrum and the neural arches; rather, premature mineralization was always contiguous with the major ossifying zones of the centrum and neural arches and their appearance coincided with the first detectable *Dlx5*-mediated expansion of the mineralized zones of centra at E17.5. Moreover, although prehypertrophic and hypertrophic zones were expanded in transgenic mice, these zones remained discrete and well defined. We did not observe, for example, isolated cells or patches of cells that were either *Ihh*- or *Col10a1*-positive within the proliferative zone of limb bones. This is in agreement with the observations of Chin *et al*. (7). In common with *Runx2* then, *Dlx5* accelerates chondrocyte differentiation but, in contrast, while *Runx2* is capable of initiating chondrocyte hypertrophy *de novo*, *Dlx5* is unable to induce ectopic chondrocyte hypertrophy in tissues where it would not normally occur, nor advance the initial timing of mineralization. Thus, it is likely that Dlx5 requires the synthesis of other rate-limiting critical cofactors for its hypertrophic function. This is an interesting situation given that Dlx5 appears to be a direct upstream regulator of *Runx2* during osteoblast differentiation [51], [52]. Nevertheless, our data strongly suggest that Dlx5 is not sufficient for transactivation of *Runx2* in chondroblasts. Indeed, the signals and upstream transcriptional regulators of *Runx2* in chondrocytes are not yet known.

### The Mosaic Nature of the Skeleton

The differential sensitivity of the appendicular and axial skeleton to a given level of ectopic *Dlx5* expression is not without precedent; non-uniform effects of *Col2a1*-driven transgenes in the endochondral skeleton have been a hallmark of other studies too. For example, the supraoccipital bone and vertebral skeleton was more sensitive to the effects of a dominant negative PTHrP receptor than the limbs, which only exhibited an effect at the highest number of transgene copies and then only in discrete elements [53]. Furthermore, expression of a constitutively active form of Akt in immature chondrocytes led to accelerated chondrocyte hypertrophy and mineralization in the cranial and vertebral skeleton but delayed hypertrophy in limbs [54]. The differential responses of the axial and appendicular skeleton to *Dlx5* and other regulators of cartilage development no doubt reflects differences in the molecular milieu of anatomically distinct chondrocytes and further underscores the mosaic nature of the skeleton.

## Materials and Methods

### Ethics Statement

Prior institutional approval was received for the animal work described in this study from the University of Guelph Animal Care Committee, in accordance with Canadian Council on Animal Care guidelines.

### Generation of Transgenic Mice, Genotyping, and RT-PCR

The murine *Dlx5* open reading frame was amplified as a 5′ *Flag*-tagged *Hin*dIII-*Nco*I fragment, and shuttled into a modified *pSlax13* [55]. The bovine polyadenylation (*bpA*) sequence from *p3000i3020Col2α1* [56] was amplified and cloned downstream of *Dlx5* as an *Nco*I-*Xba*I fragment, and the *FlagDlx5-bpA* cassette was cloned back into *p3000i3020Col2α1* as a *Hin*dIII-*Sal*I fragment, replacing the *βgeo*-*bpA* sequences. The construct was digested with *Not*I and *Sal*I and the linear transgene cassette was microinjected into the pronuclei of fertilized CD-1 oocytes. Genotyping of transgene bearing mice was done with forward primer 5′-AACAGTTCCCCGAAAGAGGT-3′ and reverse primer 5′-GAGCGCTTTGCCATAAGAAG-3′, which amplified a 1040 bp fragment. The *Col2a1-Dlx5^t19^* allele was maintained in a hemizygous state on a CD-1 background; hemizygous animals were occasionally bred to generate homozygous embryos or neonates. Offspring from *Col2a1-Dlx5^t19/+^* x *Dlx5/6^+/−^* crosses were backcrossed to *Dlx5/6* heterozygotes to generate transgene-positive, *Dlx5/6* null mice. A 438 bp genomic *Ihh* fragment was amplified with primers: 5-CACTTGTGGTGGAGGATGTG-3′ and 5′-TACCACACGCTTGTCAGCTC-3′. *Dlx5/6* heterozygotes were genotyped with the *lacZ* primer pair: 5′-GCGTTACCCAACTTAATCG-3′ and 5′-TGTGAGCGAGTAACAACC-3′ [32]. The presence of *FlagDlx5* and *β*-*actin* mRNA was determined on total RNA prepared from E16.5 embryos using the Trizol Reagent (Invitrogen). 1 µg of RNA was reverse transcribed (Superscript II, Invitrogen) and PCR amplified (Taq, UBI Life Sciences) with standard protocols. Primer sequences used for RT-PCR were: *FlagDlx5* (For 5′-GACTACAAGGACGACGATGAC-3′ and Rev 5′-GAGCGCTTTGCCATAAGAAG-3′) and *β* -*actin* (For 5′-GAGAAAATCTGGCACCACACC-3′; Rev 5′-CAGGAAGGAAGGCTGGAAGAG-3′).

### Skeletal Staining

Alcian Blue and Alizarin Red staining was used to visualize cartilaginous and mineralised skeletal tissues respectively of embryos and neonates. Briefly, eviscerated and skinned (>E16.5) bodies were fixed in 95% ethanol over several days. Embryos were stained in 80% ethanol, 20% acetic acid, 0.01% Alcian blue 8GX for 2–4 days, cleared overnight in 1% KOH, stained 1–2 days in 0.001% Alizarin Red S in 1% KOH, then rinsed in 1% KOH, and a graded series of H_2_O/glycerol to 100% glycerol.

### Histology

Tissues were fixed overnight in 4% paraformaldehyde then dehydrated and embedded in paraffin using standard techniques. 7 µm sections were stained with hematoxylin and eosin using a standard protocol.

### *In Situ* Hybridisation

Whole mount and cryosection *in situ* hybridisation was done as described previously [55] with the following antisense riboprobes: *Dlx5*, a 0.87 kb *Bam*HI – *Hin*dIII fragment corresponding to the full length open reading frame [28]; *Col2a1*, a 0.4 kb cDNA corresponding to nt 1–402 of Genbank X57982; *Ihh*, a 1.8 kb partial cDNA *Eco*RI fragment [57]; and *Col10a1*, a 0.86 kb *Apa*I - *Sal*I fragment containing nt 1351–2215 of Genbank X65121.

### Imaging

Images were taken using a MicroPublisher colour digital camera on a Leica MZ12.5 Stereomicroscope with Qcapture software (QImaging) or on a Leica DMRA2 upright microscope with Openlab software (Improvision) and processed using Adobe Photoshop.
